# Risk factors and preliminary prediction models for trauma-induced coagulopathy and 28-day mortality in severely injured patients: a retrospective observational cohort study

**DOI:** 10.3389/fmed.2026.1763737

**Published:** 2026-05-13

**Authors:** Siqi Cui, Yaru Dong, Yueguang Chen, Zhichao Jiang, Haitao Wang, Zhuning Chen, Ze Jiang, Zhaoyang Zhang

**Affiliations:** 1 Department of Scientific Research, Qilu Hospital of Shandong University, Jinan, Shandong, China; 2 Department of Emergency Surgery, Qilu Hospital of Shandong University, Jinan, Shandong, China

**Keywords:** Injury Severity Score, mortality, prediction model, Shock Index, trauma-induced coagulopathy

## Abstract

**Background:**

Trauma-Induced Coagulopathy (TIC) is a critical complication in severe trauma patients associated with high mortality. Early identification is paramount for survival, yet reliable early warning indicators remain underutilized. This study aimed to identify independent predictors for TIC and mortality, and to establish robust prediction models to guide clinical decision-making.

**Methods:**

A retrospective analysis was conducted on 128 severe trauma patients admitted to a single center. Patients were stratified into TIC (*n* = 44) and Non-TIC (*n* = 84) groups based on admission coagulation profiles. Univariate and multivariable logistic regression analyses were performed to identify risk factors. Predictive performances of the models for TIC onset and 28-day all-cause mortality were evaluated using the Area Under the Curve (AUC).

**Results:**

The incidence of TIC was 34.4%. Multivariable analysis identified Shock Index (SI), time from injury to admission, Injury Severity Score (ISS), and Glasgow Coma Scale (GCS) as independent predictors of TIC. The TIC prediction model demonstrated good discriminative ability with an AUC of 0.825. Optimal cut-off values were identified as SI > 0.73 and time from injury > 23 min, serving as early warning thresholds. Furthermore, TIC was confirmed as a potent independent risk factor for mortality (OR = 5.317, 95% CI: 1.491–18.961). The prognostic model, incorporating TIC, shock status, ISS, and GCS, achieved excellent accuracy in predicting 28-day mortality with an AUC of 0.952.

**Conclusion:**

SI, pre-hospital time, ISS, and GCS are robust early predictors of TIC. Specifically, an SI > 0.73 and a pre-hospital time exceeding 23 min serve as critical early warning thresholds warranting immediate coagulation assessment and potential intervention. The developed models provide clinicians with practical tools for early risk stratification and mortality prediction, potentially improving outcomes in severe trauma management.

## Introduction

With accelerated industrialization and transportation network development, acute trauma has emerged as a major global public health challenge (1). Epidemiological data from China indicate persistently rising trauma-related disability and mortality rates, currently ranking as the fifth leading cause of all-cause mortality and the primary cause of death among young adults aged 15–44 years (2, 3). This trend imposes substantial socioeconomic burdens through the depletion of the productive workforce. Although recent advancements in prehospital care systems, damage control surgical techniques, and critical care have significantly improved overall outcomes in trauma patients (4, 5), Trauma-Induced Coagulopathy (TIC)—a state of early coagulopathic decompensation following severe trauma—remains a critical impediment to improving survival rates (6, 7).

Current evidence indicates that approximately one-third of severe trauma patients develop TIC, with 25–36% exhibiting coagulopathy upon emergency admission (8, 9). Given the dynamic and rapid pathophysiological evolution of Trauma-Induced Coagulopathy (TIC), early and accurate identification is vital to initiate timely hemostatic interventions and improve patient outcomes. However, conventional coagulation tests (e.g., PT, APTT) frequently fail to reflect real-time *in vivo* coagulation status due to significant analytical delays (10), causing many patients to miss the critical early therapeutic window (11). Several predictive scoring systems have been developed to identify TIC early, such as the Trauma-Induced Coagulopathy Clinical Score (TICCS) (12), the Prediction of Acute Coagulopathy of Trauma (PACT) score (13), and the COAST score (14). While these tools have improved risk stratification, their clinical utility in the hyper-acute phase is often limited. Many rely on weighted variables requiring complex calculations that are impractical during resuscitation, or depend on laboratory parameters (e.g., lactate, base excess) and imaging results (CT scans) that may not be immediately available in resource-limited settings (15). Furthermore, the applicability of these models—often derived from Western military or civilian databases—to the specific injury patterns and demographics of the Chinese population remains insufficiently validated (16). Few models have integrated the Shock Index (SI)—a simple yet sensitive marker of hemodynamic instability—as a core predictor combined with injury time. Therefore, this study aimed to develop a simplified, readily available prediction model based on SI, injury time, and basic anatomical scores to facilitate early decision-making for severe trauma patients.

Addressing these limitations, there is a clear need for a more pragmatic screening approach that relies on universally available clinical metrics. Therefore, this study aims to: (1) Identify independent risk factors for TIC and 28-day mortality in severe trauma patients; and (2) Construct and validate a simplified prediction model based on readily accessible indicators—specifically the Injury Severity Score (ISS), Glasgow Coma Scale (GCS), and injury time. This study seeks to provide a practical tool for the rapid identification of high-risk cohorts, particularly suitable for emergency scenarios where advanced diagnostic resources are limited or delayed.

## Methods

### Study design and settings

This study was designed as a retrospective observational cohort study and is reported in accordance with the STROBE (Strengthening the Reporting of Observational Studies in Epidemiology) statement and the TRIPOD (Transparent Reporting of a multivariable prediction model for Individual Prognosis Or Diagnosis) guidelines. We screened all consecutive trauma patients admitted to the Emergency Department (ED) between April 19, 2022, and April 18, 2023. The study protocol was approved by the Institutional Review Board (no. KYLL202203-031-1). Due to the retrospective nature of the study, the requirement for informed consent was waived by the Ethics Committee. All procedures complied with the Declaration of Helsinki. To ensure data privacy, all personal identifiers were removed upon data extraction.

**Figure 1:**
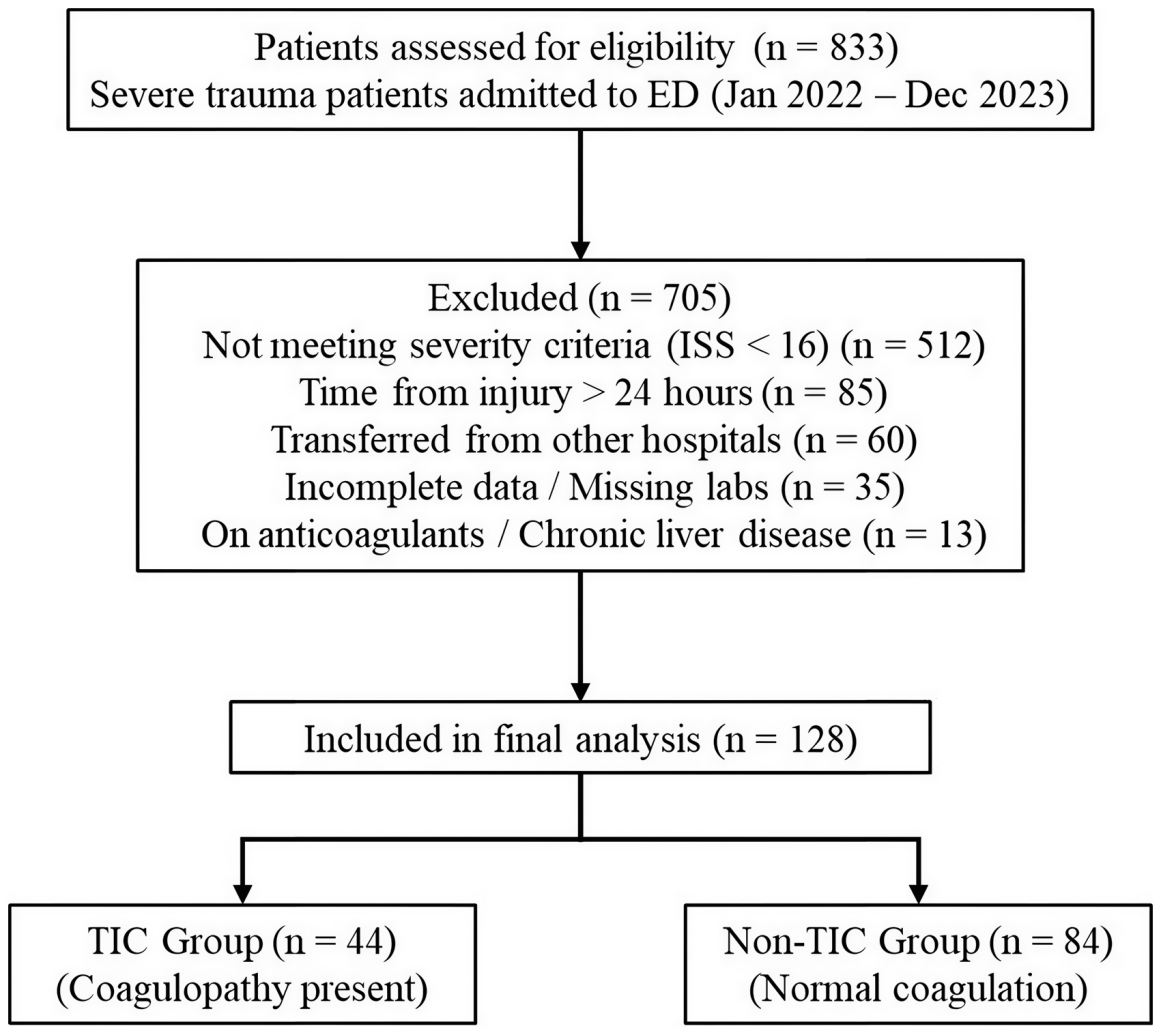
The diagram illustrates the inclusion and exclusion process for the study cohort based on STROBE guidelines. A total of 833 trauma patients presented to the Emergency Department between January 2022 and December 2023. After applying exclusion criteria—specifically excluding patients with minor injuries (ISS < 16) and delayed admission (> 24 hours)—128 severely injured patients were included in the final analysis and stratified into TIC (*n* = 44) and Non-TIC (*n* = 84) groups. ISS, Injury Severity Score; TIC, Trauma-Induced Coagulopathy; ED, Emergency Department.

Participants and Eligibility A total of 833 trauma patients were initially screened. A flow diagram of patient selection is presented in Figure 1. Inclusion criteria: (1) Age ≥18 years; (2) History of acute trauma; (3) Severe trauma, defined as an Injury Severity Score (ISS) ≥ 16; (4) Admission to the ED within 24 h of injury. Exclusion criteria: (1) Incomplete medical records (missing critical data on coagulopathy or outcomes); (2) Use of anticoagulants or antiplatelet agents within 7 days prior to injury; (3) Known pre-existing coagulation disorders (e.g., hemophilia, liver cirrhosis); (4) Inter-hospital transfers (to avoid confounding by pre-transfer interventions); (5) Dead on arrival or death before initial blood sampling.

Data Collection and Definitions Data were extracted from the Electronic Medical Record (EMR) system by two trained investigators using a standardized data collection form. Discordances were resolved by a third senior reviewer. 1. Clinical Variables: Demographic data (age, sex, BMI), comorbidities, injury mechanism (blunt vs. penetrating), and vital signs upon ED arrival (systolic blood pressure [SBP], heart rate, temperature) were recorded.

Shock was defined as a Shock Index (SI) > 1.0 (calculated as Heart Rate/SBP).
Hypothermia was defined as a body temperature < 35.0 °C measured upon admission.

#### Injury severity

The Glasgow Coma Scale (GCS) was assessed prior to sedation or intubation. The ISS was calculated based on the Abbreviated Injury Scale (AIS) 2005 (2008 update). While ISS is calculated retrospectively, it serves as a proxy for the anatomical injury burden present at admission.

### Outcome definitions

#### Trauma-Induced Coagulopathy (TIC)

TIC was defined based on the initial laboratory values obtained immediately upon ED admission (prior to significant fluid resuscitation or transfusion). Consistent with international guidelines and established criteria, TIC was diagnosed if any of the following thresholds were met: International Normalized Ratio (INR) > 1.5, Activated Partial Thromboplastin Time (APTT) > 35 s, or Fibrinogen < 1.5 g/L.

#### Mortality

The secondary outcome was 28-day all-cause mortality, confirmed via hospital records or follow-up calls.

Software and Data Handling Statistical analyses were performed using SPSS version 26.0 (IBM Corp, Armonk, NY) and R software (version 4.2.0; R Foundation for Statistical Computing, Vienna, Austria). Variables with >10% missingness were excluded from the primary analysis. Missing data for included covariates were assumed to be missing at random (MAR) and addressed using Multiple Imputation by Chained Equations (MICE) to generate five imputed datasets.

Descriptive Statistics Data normality was assessed using the Shapiro–Wilk test. Continuous variables are expressed as mean ± standard deviation (SD) or median [interquartile range, IQR] and compared using Student’s t-test or the Mann–Whitney U test, as appropriate. Categorical variables are reported as frequencies (*n*, %) and compared using the Chi-square test or Fisher’s exact test.

Model Development and Variable Selection to identify independent predictors for TIC and 28-day mortality, potential candidates were initially screened via univariate analysis. Variables demonstrating a *p*-value < 0.10 in Table 1 (e.g., pulse rate, Shock Index, GCS, and ISS) or those deemed clinically essential based on existing trauma literature were considered for multivariable logistic regression.

**Table 1:** Comparison of clinical parameters and related test indices between the TIC and non-TIC groups.

Risk factors		Non-TIC groups (*n* = 84)	TIC groups (*n* = 44)	Test value, *t/Z/χ^2^*	*p* value
Genders	Male	70 [83.3]	31[70.5]	2.877	0.090
	Female	14 [16.7]	13[29.5]		
Age		47.42 ± 13.51	46.20 ± 15.60	0.457	0.649
Causes of trauma	Traffic accident	20 [23.8]	7[15.9]	4.333	0.222
	Fall from height	33 [39.3]	18[40.9]		
	Mechanical injury	5 [6.0]	0[0]		
	Others	26 [31.0]	19[43.2]		
Body temperature		36.83 ± 0.72	36.40 ± 0.86	2.063	0.153
Pulse rate		86.65 ± 17.17	101.61 ± 22.31	4.432	0.037^*^
Shock Index		0.70 ± 0.17	0.86 ± 0.26	7.183	0.008^*^
GCS score		15.00 [13.00, 15.00]	12.00 [7.00, 15.00]	−3.23	<0.001^*^
ISS score		18.00 [16.00, 21.75]	25.00 [18.00, 29.00]	4.815	<0.001^*^
Time from injury at admission		24.00 [10.00, 24.00]	13.00 [7.00, 24.00]	−2.239	0.025^*^
PT		13.75 [12.42, 14.50]	19.85 [19.32, 23.55]	8.640	<0.001^*^
APTT		29.70 [26.80, 33.47]	64.10 [60.60, 67.75]	8.602	<0.001^*^
INR		1.08 [1.00, 1.14]	1.59 [1.50, 1.66]	8.797	<0.001^*^
Fib		2.98 [2.52, 3.88]	1.41 [1.01, 2.10]	−7.877	<0.001^*^
FDP		17.40 [9.23, 35.18]	30.89 [11.20, 102.53]	2.044	0.041^*^
D-D		4.36 [2.21, 11.56]	8.38 [3.09, 15.78]	1.784	0.074
WBC		10.91 [8.94, 14.77]	9.56 [7.64, 11.58]	−1.999	0.046^*^
NEUT		87.50 [82.75, 91.60]	86.80 [77.87, 90.10]	−1.515	0.130
RBC		3.69 [3.16, 4.30]	3.56 [2.98, 4.27]	−0.715	0.475
HCT		34.50 [29.43, 41.20]	32.95 [27.40, 38.80]	−0.903	0.366
HB		115.00 [98.25, 137.00]	111.00 [91.75, 132.25]	−0.522	0.602
PLT		176.00 [138.00, 227.00]	124.00 [82.50, 161.25]	−4.909	<0.001
PCT		0.17 [0.14, 0.21]	0.17 [0.14, 0.20]	−0.055	0.956

*p < 0.05, indicating statistical significance.

To ensure robust predictor selection and mitigate the risk of overfitting—given the study cohort (*n* = 128) and the number of events (44 TIC cases, 27 deaths)—we employed a backward stepwise selection process based on the likelihood ratio. While we acknowledge that maintaining four critical predictors (SI, time from injury, ISS, and GCS) slightly stretched the traditional “10 events per variable” (EPV) rule of thumb, these variables were purposefully retained in the final models due to their established physiological relevance in trauma-induced coagulopathy and survival. We acknowledge that the strong association between SI and outcomes, combined with the modest sample size, resulted in wide confidence intervals in some models; however, these were interpreted primarily as indicators of high effect size within this specific cohort rather than fundamental model instability. To account for this, our models are presented as exploratory tools requiring further external validation in larger, multi-center cohorts.

#### Model performance and validation

Model discrimination was evaluated using the Area Under the Receiver Operating Characteristic Curve (AUC). Calibration was assessed using the Hosmer-Lemeshow test and visual calibration plots. To address potential optimism bias and ensure the reliability of the prognostic estimates, internal validation was performed using bootstrapping with 1,000 resamples to calculate the optimism-corrected AUC and 95% confidence intervals. Multicollinearity was assessed using Variance Inflation Factors (VIF), with a threshold of < 5 indicating no significant collinearity. A two-tailed *p*-value < 0.05 was considered statistically significant.

## Results

### Patient characteristics and univariate analysis

A total of 128 severe trauma patients (ISS ≥ 16) were included in the final analysis. The incidence of Trauma-Induced Coagulopathy (TIC) in this cohort was 34.4% (*n* = 44). Compared to the non-TIC group (*n* = 84), patients who developed TIC presented with significantly higher Injury Severity Scores (ISS) and lower Glasgow Coma Scale (GCS) scores upon admission (*p* < 0.05). Notably, univariate analysis (Table 1) revealed that patients in the TIC group exhibited significantly higher pulse rates (101.61 ± 22.31 vs. 86.65 ± 17.17, *p* = 0.037) and higher Shock Index values (0.86 ± 0.26 vs. 0.70 ± 0.17, *p* = 0.008). Furthermore, the TIC group had a significantly longer pre-hospital time (time from injury to admission) compared to those without coagulopathy. These significant variables (*p* < 0.10) were subsequently advanced to the multivariable modeling stage.

### Independent risk factors for TIC development

A multivariable logistic regression model was constructed to identify independent predictors of TIC. The model demonstrated good goodness-of-fit (Hosmer-Lemeshow test, χ^2^ = 3.636, *p* = 0.888) and explained a substantial proportion of the variance (Nagelkerke R^2^ = 0.536). As shown in Table 2, after adjusting for potential confounders, four variables were identified as independent risk factors for TIC:

**Table 2:** Multivariate logistic regression analysis for predictors of TIC.

Variables	*β*	S. E.	Wald χ^2^	*p*-value	OR	95% CI for OR
Shock Index	2.669	1.151	5.377	0.020	14.420	1.511–137.574
Time from injury	0.032	0.014	5.606	0.018	1.033	1.006–1.060
GCS classification						
Severe vs. Non-severe	1.271	0.485	6.873	0.009	3.565	1.378–9.220
ISS classification						
Severe vs. Non-severe	1.581	0.479	10.900	0.001	4.861	1.901–12.427
Constant	−4.502	0.992	20.600	<0.001	0.011	—

#### Shock Index (SI)

Higher SI was strongly associated with TIC onset (OR = 14.420, 95% CI: 1.511–137.574, *p* = 0.020).

#### Time from injury

Delayed admission was a significant predictor (OR = 1.033, 95% CI: 1.006–1.060, *p* = 0.018), confirming that prolonged pre-hospital time increases coagulopathy risk.

#### ISS and GCS

Severe anatomical injury (ISS) and depressed consciousness (GCS) remained significant independent predictors. Specifically, severe ISS classification (OR = 4.861, 95% CI: 1.901–12.427, *p* = 0.001) and severe GCS classification (OR = 3.565, 95% CI: 1.378–9.220, *p* = 0.009) strongly predicted the development of TIC.

### Performance of the TIC prediction model and optimal cut-off values

**Figure 2:**
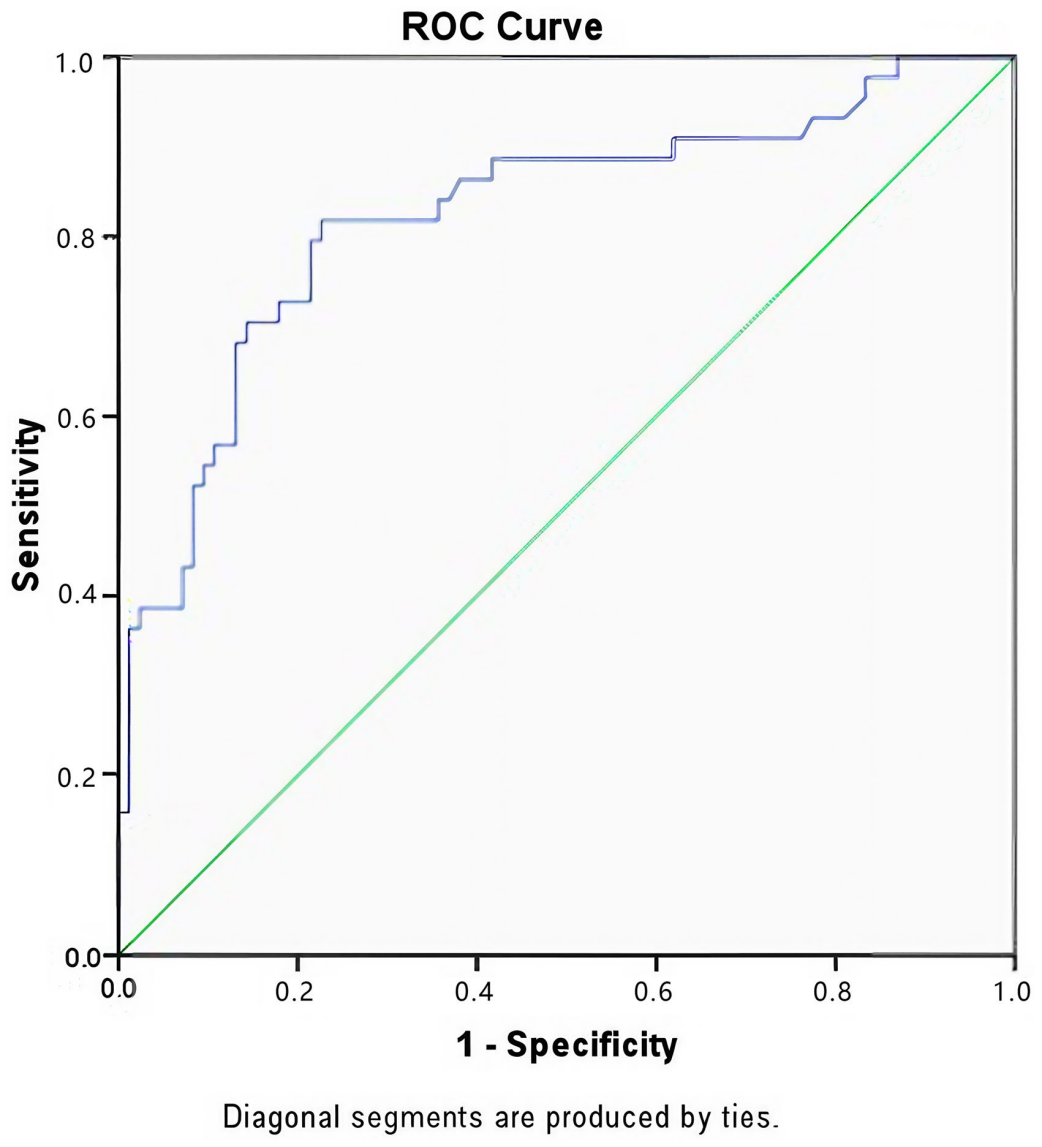
Receiver operating characteristic (ROC) curve for the prediction of Trauma-Induced Coagulopathy (TIC). The multivariate model (incorporating Shock Index, time from injury, ISS, and GCS) demonstrated good discriminative ability with an AUC of 0.825 (*p* < 0.001).

The discriminative ability of the TIC prediction model was evaluated using Receiver Operating Characteristic (ROC) curve analysis. The multivariable model (combining SI, Time from Injury, ISS, and GCS) achieved an Area Under the Curve (AUC) of 0.825 (95% CI: 0.750–0.900, *p* < 0.001), indicating excellent predictive performance (Figure 2). To provide practical clinical guidance, optimal cut-off values for the continuous predictors within this cohort were determined using the Youden Index. The optimal cut-off for Shock Index was > 0.73 (Sensitivity: 70.5%, Specificity: 61.9%), suggesting that an admission SI exceeding this threshold should trigger immediate coagulation monitoring. For Time from Injury, a threshold of > 23 min was identified (sensitivity: 70.5%, specificity: 58.3%), emphasizing the critical “golden window” for early intervention in severe trauma.

### Predictors of mortality and prognostic model

**Figure 3:**
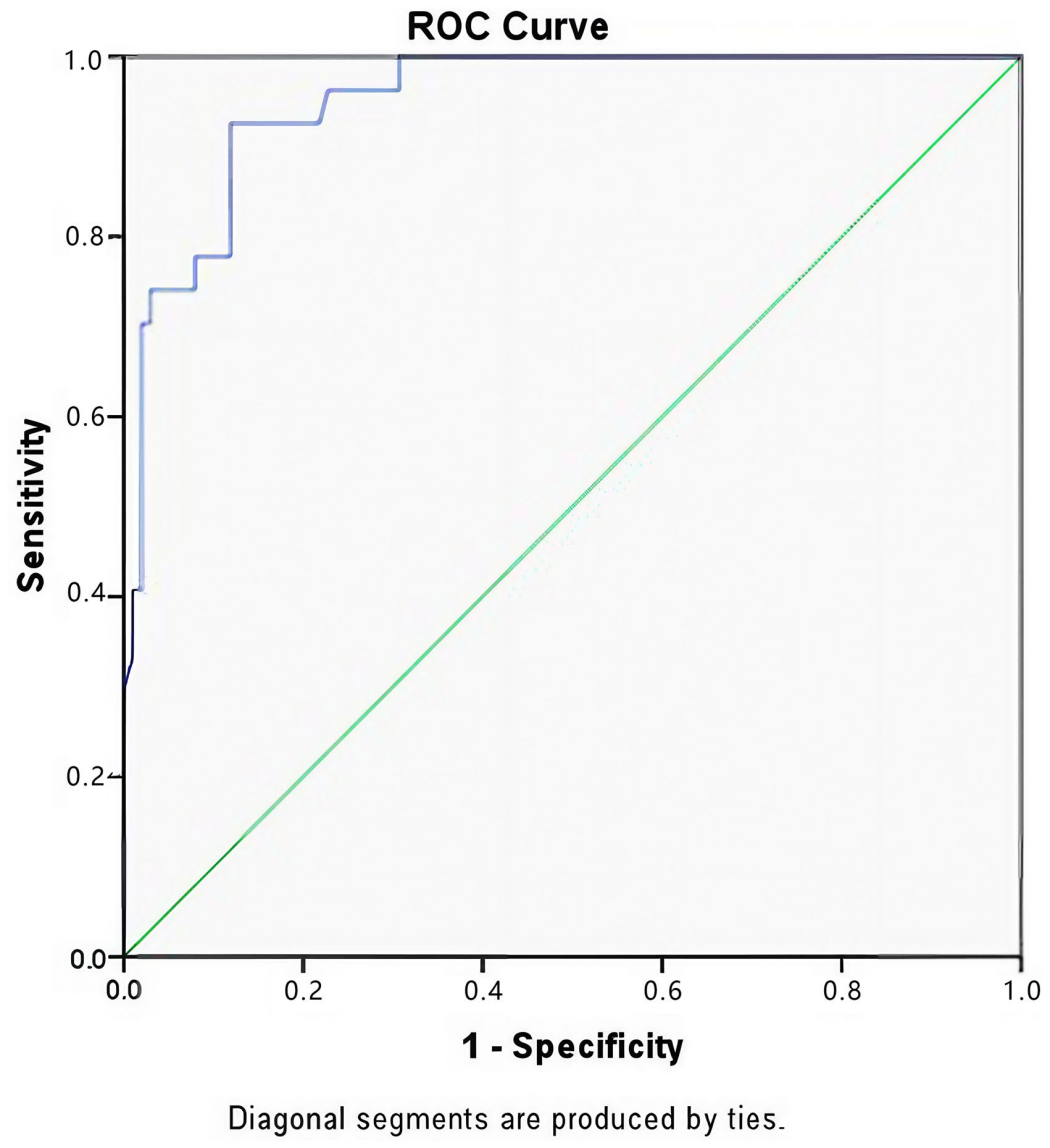
Receiver operating characteristic (ROC) curve for the prediction of 28-day mortality. The prognostic model (incorporating TIC presence, Shock Index, ISS, and GCS) showed excellent performance with an AUC of 0.952 (*p* < 0.001), indicating high accuracy in distinguishing survivors from non-survivors.

The overall mortality rate in the study cohort was 21.1% (*n* = 27). A second multivariable logistic regression analysis was performed to determine the impact of TIC on survival, adjusting for injury severity and shock. The model showed exceptional fit (Nagelkerke R^2^ = 0.688; Hosmer-Lemeshow test *p* = 0.898). Trauma-Induced Coagulopathy was identified as an independent predictor of mortality. Patients with TIC had an over 5-fold increase in the risk of death compared to those without TIC (OR = 5.317, 95% CI: 1.491–18.961, *p* = 0.010). Other robust independent predictors of mortality included the presence of shock upon admission (OR = 15.754, 95% CI: 2.852–87.017, *p* = 0.002) and severe ISS classification (OR = 8.505, 95% CI: 2.310–31.314, *p* = 0.001). While severe GCS classification was retained in the final model due to its clinical relevance, it showed a trend toward significance in predicting mortality (OR = 2.835, 95% CI: 0.834–9.638, *p* = 0.095) (Table 3). The prognostic model for mortality demonstrated outstanding discriminative power, with an AUC of 0.952 (95% CI: 0.910–0.990, *p* < 0.001) (Figure 3). This indicates that the combination of TIC presence, hemodynamic status, and injury severity can accurately predict outcomes in severe trauma patients.

**Table 3:** Multivariate logistic regression analysis for predictors of 28-day mortality.

Variables	*β*	S. E.	Wald χ^2^	*p*-value	OR (95% CI)
Shock (yes vs. no)	2.757	0.872	9.998	0.002	15.754 (2.852–87.017)
TIC (yes vs. no)	1.671	0.649	6.635	0.010	5.317 (1.491–18.961)
ISS classification^a	2.141	0.665	10.363	0.001	8.505 (2.310–31.314)
GCS classification^b	1.042	0.624	2.785	0.095	2.835 (0.834–9.638)
Constant	−4.102	0.704	33.936	<0.001	—

## Discussion

In this study, we identified the independent risk factors for Trauma-Induced Coagulopathy (TIC) and mortality in a cohort of 128 severe trauma patients. A key contribution of our work is the development of two high-performance prediction models. The TIC prediction model, incorporating Shock Index (SI), time from injury, ISS, and GCS, achieved an AUC of 0.825, demonstrating excellent discriminative ability. Furthermore, we confirmed that TIC is a potent independent predictor of mortality (OR ≈ 5.3), and our prognostic model predicted 28-day survival with remarkable accuracy (AUC = 0.952). These findings provide clinicians with tangible, evidence-based tools for early risk stratification.

Hemodynamic instability is a well-established driver of coagulopathy, primarily through the mechanisms of hypoperfusion and Protein C activation (17, 18). Our study reinforces the superiority of the Shock Index (SI) over traditional vital signs. While heart rate and blood pressure can be temporarily compensated in the early stages of shock, SI serves as a more sensitive unmasking indicator (19). Notably, our ROC analysis identified an optimal SI cut-off value of 0.73. Since the normal range of SI is typically 0.5–0.7 (20), our data suggest that once the SI exceeds this upper physiological limit, the risk of TIC rises exponentially. This provides a simple, immediate bedside “red flag” for trauma teams: any admission SI > 0.73 warrants immediate coagulation testing and potential prophylactic intervention.

The adage “time is blood” remains central to trauma care. Our multivariable analysis confirmed that prolonged time from injury to admission is an independent risk factor for TIC (21, 22). Interestingly, our threshold analysis revealed a sensitive cut-off time of > 23 min. While this duration is relatively short, it reflects the rapid onset of acute traumatic coagulopathy (ATC) driven by tissue injury and shock, rather than mere dilution from resuscitation (23). This finding underscores the critical importance of pre-hospital efficiency. For patients with severe trauma, a pre-hospital delay exceeding this short window may be sufficient to initiate the cascade of coagulopathy, necessitating “scoop and run” strategies and potentially early administration of tranexamic acid.

Consistent with previous literature, higher ISS and lower GCS scores were strongly associated with TIC. Severe tissue damage leads to a massive release of tissue factor and inflammatory cytokines, triggering systemic coagulation activation (24). Similarly, the significant association between low GCS and TIC highlights the unique pathophysiology of traumatic brain injury (TBI)-induced coagulopathy (25, 26). While our model demonstrates the predictive value of these variables, we acknowledge the practical challenges of definitively calculating the ISS in the hyper-acute phase, as it typically relies on comprehensive imaging (e.g., whole-body CT scans). However, in real-world resuscitation, experienced clinicians routinely estimate the anatomical injury burden during the primary survey (via injury mechanism, physical examination, eFAST, etc.). Therefore, in the context of our early prediction model, the “severe ISS” classification should be interpreted as a proxy for clinically evident severe anatomical trauma upon presentation, complementing the immediately available SI and GCS to guide early blood product preparation.

Perhaps the most sobering finding of our study is the profound impact of TIC on survival. Patients who developed TIC had an over 5-fold increased risk of death compared to those who did not, independent of their injury severity. This aligns with the concept of the “Lethal Triad” (27). Furthermore, our mortality prediction model achieved an exceptional AUC of 0.952, implying that 28-day survival is highly predictable based on the combination of coagulopathy (TIC), the presence of shock, and injury burden (ISS and GCS). This highlights that the early correction of TIC and the rapid reversal of shock are the most modifiable targets to improve survival rates.

Regarding the specific optimal cut-off values identified (SI > 0.73 and Time from injury > 23 min), we must emphasize that these thresholds are inherently derived from the specific demographics, geographic distribution, and trauma system characteristics of our single-center cohort. Rather than serving as universal absolutes, these cut-offs highlight a conceptual “golden window”—demonstrating that even mild early hemodynamic instability, when combined with prolonged pre-hospital time, significantly amplifies the risk of coagulopathy. Future multi-center prospective studies are required to validate and recalibrate these specific thresholds across different populations.

Limitations This study has several important limitations that must be acknowledged. First and foremost is the relatively small sample size (*n* = 128) and the limited number of TIC events (*n* = 44). Consequently, maintaining four critical predictors (SI, Time, GCS, ISS) in our multivariable logistic regression model slightly stretched the strict “10 events per variable” (EPV) rule of thumb. This constraint likely contributed to the wide 95% confidence interval observed for the Shock Index (95% CI: 1.511–137.574). While the large Odds Ratio reflects a strong underlying physiological association between profound shock and coagulopathy, the wide interval indicates a degree of statistical uncertainty regarding the precise effect size. Second, although we attempted to mitigate the risk of overfitting by employing backward stepwise selection and performing internal validation via bootstrapping (1,000 resamples), the potential for optimism bias remains in models derived from small cohorts. Therefore, the current prediction models should be viewed as exploratory and hypothesis-generating. Third, as a retrospective observational study, it is subject to inherent biases, including missing data and unmeasured confounders (e.g., pre-hospital fluid administration volumes and specific pre-hospital interventions), which could impact coagulation status upon admission.

Lastly, due to the single-center design, extensive external validation in larger, multi-center, and prospective cohorts is mandatory to confirm the models’ stability, reproducibility, and generalizability before they can be routinely implemented in clinical practice.

## Conclusion

Shock Index (SI), time from injury, Injury Severity Score (ISS), and Glasgow Coma Scale (GCS) are critical early predictors of Trauma-Induced Coagulopathy (TIC). This study successfully developed a risk prediction model with excellent discriminative ability (AUC = 0.825). Specifically, a Shock Index > 0.73 and an admission time > 23 min serve as critical early warning thresholds for coagulopathy onset. Furthermore, TIC is a potent independent risk factor for mortality, and our prognostic model demonstrated outstanding accuracy (AUC = 0.952) in predicting fatal outcomes. These findings highlight the potential of using these readily available admission parameters for early risk stratification and targeted coagulation management, though large-scale prospective validation is warranted prior to routine clinical implementation.

## Data Availability

The raw data supporting the conclusions of this article will be made available by the authors, without undue reservation.
